# Developmental shifts in testosterone levels are associated with alterations in the neural oscillatory dynamics serving selective attention

**DOI:** 10.1162/IMAG.a.120

**Published:** 2025-08-26

**Authors:** Jake J. Son, Abraham D. Killanin, Camilo A. Castelblanco-Riveros, Seth D. Springer, Hallie J. Johnson, Hannah J. Okelberry, Lucas Weyrich, Yu Ping Wang, Vince D. Calhoun, Julia M. Stephen, Brittany K. Taylor, Giorgia Picci, Tony W. Wilson

**Affiliations:** Institute for Human Neuroscience, Boys Town National Research Hospital, Boys Town, NE, United States; Center for Pediatric Brain Health, Boys Town National Research Hospital, Boys Town, NE, United States; College of Medicine, University of Nebraska Medical Center, Omaha, NE, United States; Department of Psychology and Brain Sciences, Dartmouth College, Hanover, NH, United States; Department of Neurology, Dartmouth-Hitchcock Medical Center, Lebanon, NH, United States; Department of Biomedical Engineering, Tulane University, New Orleans, LA, United States; Tri-Institutional Center for Translational Research in Neuroimaging and Data Science (TReNDS), Georgia State University, Georgia Institute of Technology, Emory University, GA, United States; Mind Research Network, Albuquerque, NM, United States; Department of Pharmacology & Neuroscience, Creighton University, Omaha, NE, United States

**Keywords:** magnetoencephalography, MEG, alpha, gamma, development, hormones

## Abstract

The transition from childhood into adolescence is associated with marked increases in testosterone, a sex hormone that has been linked with significant changes in brain structure and function. However, the majority of the extant literature on sex hormone effects has focused on structural brain development, with far fewer studies examining changes in the neural dynamics serving higher-order cognitive function and behavioral improvements with development. Herein, we investigated whether the neural oscillatory dynamics serving selective attention were sensitive to testosterone levels as a marker of development in a sample of 87 participants aged 6–13 years old. Participants completed a number-based Simon task while undergoing magnetoencephalography (MEG) and the resulting data were transformed into the time-frequency domain, imaged using a beamformer, and analyzed using whole-brain analysis of covariance models. Our key findings included spectrally-specific alterations in alpha and gamma oscillatory power in the prefrontal, parietal, and temporal regions with developmental shifts in testosterone levels, after accounting for the effect of age. Additionally, sex-by-testosterone interactions were found in the anterior cingulate, prefrontal, and parietal cortices that may indicate sexually divergent brain network development during the employment of selective attention. In sum, these results provide crucial new evidence supporting a relationship between developmental changes in testosterone and functional brain dynamics in youth during a critical period for skill acquisition and refinement.

## 1 Introduction

Puberty is a critical period of major physical, cognitive, and emotional change, thought to be induced in part by the increased production of sex hormones in conjunction with exposure to novel environments and social and affective contexts. Specifically, increases in testosterone during puberty have been linked with functional and structural brain development (Ahmed et al., 2008; Blakemore et al., 2010; Goddings et al., 2014; Sisk et al., 2003; Vijayakumar et al., 2018). These alterations (e.g., cortical thinning) occur alongside physical and behavioral changes in both females and males, such as the development of secondary sex characteristics and increased social maturation and competency (Choudhury, 2010; Dahl, 2004; Forbes & Dahl, 2010). The integration of pubertal hormone indices and human neuroimaging is a rapidly growing area, with studies to date mainly focusing on the linkage between pubertal hormone levels and developmental changes in brain structure (i.e., cortical thickness and/or volume; Bramen et al., 2012; Giedd et al., 1999; Herting et al., 2014, 2015; Herting & Sowell, 2017; Lenroot et al., 2007; Nguyen, McCracken, Ducharme, Botteron, et al., 2013; Nguyen, McCracken, Ducharme, Cropp, et al., 2013; Vijayakumar et al., 2018). Such changes in cortical thickness appear to be sex-specific, tightly linked to testosterone levels in 4–22-year-olds (Nguyen, McCracken, Ducharme, Botteron, et al., 2013), and observed across a broadly distributed network of cortical regions, including somatosensory and association cortices (Bramen et al., 2012; Koolschijn et al., 2014; Neufang et al., 2009; Nguyen, McCracken, Ducharme, Botteron, et al., 2013). For instance, Bramen et al. (2012) found that testosterone levels were tightly linked to cortical thickness in the left inferior parietal, middle temporal, and striate cortices, with sex-by-testosterone interaction effects detected in higher-order cortices. Further, Koolschijn et al. (2014) found sex-dependent effects of testosterone on cortical thinning in the anterior cingulate cortex. Importantly, many of these pubertal effects are appreciable while controlling for the effect of chronological age, indicating that while closely related, pubertal processes provide complementary information regarding developmental processes that are dissociable from age-related developmental changes (Blakemore et al., 2010; Laube et al., 2020; Vijayakumar et al., 2018). However, while these studies have contributed substantially to our understanding of the sex-dependent effects of testosterone on developmental changes in brain *structure*, there is a dearth of literature examining hormone-related changes in brain *function*. Thus, studies of testosterone throughout this sensitive developmental period may provide more nuanced insights into the impact of pubertal processes on brain development, particularly with respect to the emergence of sex differences.

The majority of existing electroencephalographic (EEG) studies evaluating the impact of testosterone on brain function focus on social-affective processes (Goddings et al., 2012; Laube et al., 2020; Tyborowska et al., 2016; Vijayakumar et al., 2018, 2019), with a relative paucity of investigations on executive functions like selective attention and working memory. However, there is increasing interest in how pubertal hormones may affect the development of such higher-order processes, including works via magnetoencephalography (MEG) focused on the neural oscillatory dynamics underlying such processes (Fung et al., 2020; Fung, Heinrichs-Graham, et al., 2022; Fung, Rahman, et al., 2022). For example, Fung et al. (2020) reported that attention-related gamma oscillations in the right temporal-parietal junction exhibited sex-specific testosterone effects above and beyond the effect of chronological age. Specifically, female youth exhibited stronger gamma responses with increasing testosterone during visuospatial attention processing, whereas male youth exhibited the opposite pattern. A similar pattern of responses was found in the parietal and occipital cortices (Fung et al., 2020). Interestingly, sex-by-testosterone interaction effects have been reported in other studies focused on neural oscillations and/or spontaneous neural activity in youth, with sex-by-testosterone effects in the prefrontal cortices within the gamma band being a key finding (Picci et al., 2023). Taken together, an emerging body of work highlights the sensitivity of gamma-frequency neural activity to testosterone levels during the development.

While the aforementioned studies showed that testosterone was associated with changes in the brain dynamics underlying visuospatial attention, motor control, and the resting-state, no studies to date have examined how testosterone may modulate selective attention, which has been studied extensively in adults and is being increasingly studied in developmental populations (McDermott et al., 2017; Rosenberg et al., 2021; Son et al., 2024; Spooner et al., 2020; Taylor et al., 2021). The Simon task, a widely used assessment of selective attention where stimulus-response incompatibility introduces a behavioral cost, has been found to elicit robust multispectral neural responses in adults, including increased gamma and alpha oscillations (Rosenberg et al., 2021; Son et al., 2023; Wiesman & Wilson, 2020a; Wiesman et al., 2020). Recently, Son et al. reported robust developmental effects in the context of the Simon task, including stronger gamma oscillations with increasing age in the dorsolateral prefrontal cortex, frontal eye fields, and supplementary motor area of typically developing children and adolescents (Son et al., 2024). However, given the robust correlation between chronological age and testosterone levels during childhood and adolescence, such developmental gamma changes serving selective attention could more closely reflect developmental increases in testosterone, but further work is needed. Testosterone metabolites act via multiple pathways and have been implicated in the direct modulation of GABAergic receptors (Celec et al., 2015; Hebbard et al., 2003; Michels & Hoppe, 2008). Specifically, testosterone can exert positive allosteric effects on GABA receptors (Aikey et al., 2002; Laube et al., 2020; McHenry et al., 2014), thereby directly impacting GABA-mediated high-frequency gamma (>30 Hz) oscillations and other types of neuronal communication (Bartos et al., 2007; Buzsáki & Wang, 2012; Edden et al., 2009; Fries et al., 2007).

The current study aimed to partially fill this gap by examining the relationship between testosterone and the neural oscillatory dynamics serving selective attention in typically developing children and adolescents. Specifically, participants completed a modified numeric Simon task while undergoing MEG. The neural oscillatory activity was then imaged and integrated with data on testosterone levels, while accounting for the effects of chronological age and saliva collection time. Based on the prior literature, we hypothesized that regions that are rich in androgen receptors and are critical for selective attention (e.g., fronto-parietal networks) would be tightly coupled to testosterone levels, above and beyond the effects of age, and in at least some cases, would differ by sex.

## 2 Methods

### 2.1 Participants

We examined 126 youth (58 female (46%)) with an age range of 6- to 13-years-old (*M* = 9.75 years, *SD* = 2.55) who were enrolled into an accelerated longitudinal study (i.e., the NIH supported Dev-MIND project) that consisted of two age groups, 6- to 8-year-olds (*M* = 7.44 years, *SD* = 0.92) and 11- to 13-year-olds (*M* = 12.24 years, *SD* = 0.87). Herein, we focus on data collected during year 1. Thus, there were no 9- or 10-year-olds, as these data would be collected in later years when the younger group reached these ages. A total of 120 participants provided salivary samples for hormonal assay during year 1 and underwent neuroimaging, and we focus on these participants. Exclusionary criteria included an inability to perform the task, any medical illness affecting central nervous system function, neurological or psychiatric disorders (e.g., autism, epilepsy), as well as the laboratory’s standard exclusion criteria for magnetic resonance imaging (MRI) and MEG scans (e.g., dental braces, metal implants, battery operated implants).

### 2.2 Ethics

Parents of children signed informed consent, and children signed informed assent before proceeding with the study. An Institutional Review Board approved all procedures and were in accordance with the Declaration of Helsinki.

### 2.3 Saliva testosterone collection and measurement

Saliva was collected via passive drool into an Oragene DISCOVER (OGR-500; www.dnagenotek.com) collection tube until liquid saliva exceeded the fill line indicated on the tube, which was 2.0 mL. Participants were instructed to refrain from consuming any food, liquids, or chewing gum for at least an hour before providing the saliva sample and generally completed the study in the afternoon (*M* = 15:01 hr, *SD* = 2:49 hr). Prior to the release of the protease inhibitors for long-term storage, a single-channel pipette was used to extract 0.5 mL from the collection tube and placed in a -20°C freezer for storage. Thus, the saliva samples were not contaminated by the preservative prior to hormonal analysis. All samples were assayed with duplicate testing using a commercially-available assay kit for salivary testosterone (Salimetrics EIA High Sensitivity Salivary Testosterone Kit; www.salimetrics.com). The assay kit had a sensitivity of 0.67 pg/mL, with a range of 6.1–600.0 pg/mL. The intra and inter-assay coefficients of variation were 5.04% and 3.31%, respectively. The arithmetic means of the duplicate tests were used for analyses in the present study. A Shapiro-Wilk test of the testosterone levels showed that the data were not normally distributed, thus a natural log transform was applied prior to further analyses ([*W*_before_ = 0.85, *p* < 0.001; *W*_after_ = 0.98, *p* = .27]).

### 2.4 MEG and MRI data acquisition

MEG data acquisition, structural coregistration, preprocessing, and sensor-/source-level analyses closely followed a standardized analysis pipeline (Spooner et al., 2019, 2021; Wiesman & Wilson, 2020b). All recordings were conducted in a one-layer magnetically shielded room with active shielding engaged. Prior to MEG measurement, four coils were attached to the participant’s head and localized together with the three fiducial points and scalp surface using a 3D digitizer (Fastrak, Polhemus Navigator Sciences, Colchester, VT, USA). Once the participant was positioned for MEG recording, an electric current with a unique frequency label (e.g., 322 Hz) was fed to each of the coils. The magnetic fields associated with these currents were then localized in correspondence to the sensors throughout the recording session. Since coil locations were also known in head coordinates, all MEG measurements could be transformed into a common coordinate system. Neuromagnetic responses were sampled continuously at 1 kHz with an acquisition bandwidth of 0.1–330 Hz, using a 306-sensor Elekta/MEGIN MEG system (Helsinki, Finland), equipped with 204 planar gradiometers and 102 magnetometers. For analysis, we only considered data from the 204 planar gradiometers. Participants were monitored during data acquisition via real-time audio-video feeds from inside the shielded room. Each participant’s data were individually corrected for head motion, and noise reduction was applied using the temporally-extended signal space separation approach (Taulu & Simola, 2006). Structural T1-weighted images were acquired using a Siemens Prisma 3T MRI scanner (Siemens Healthineers AG, Erlangen, Germany) with a 32-channel head coil and a 3D MP-RAGE sequence with the following parameters: TR = 2400 ms; TE = 2.05 ms; flip angle = 8°; FOV = 256 mm; slice thickness = 1 mm; and voxel size = 1 mm^3^.

### 2.5 The Simon task

**Fig. 1.**
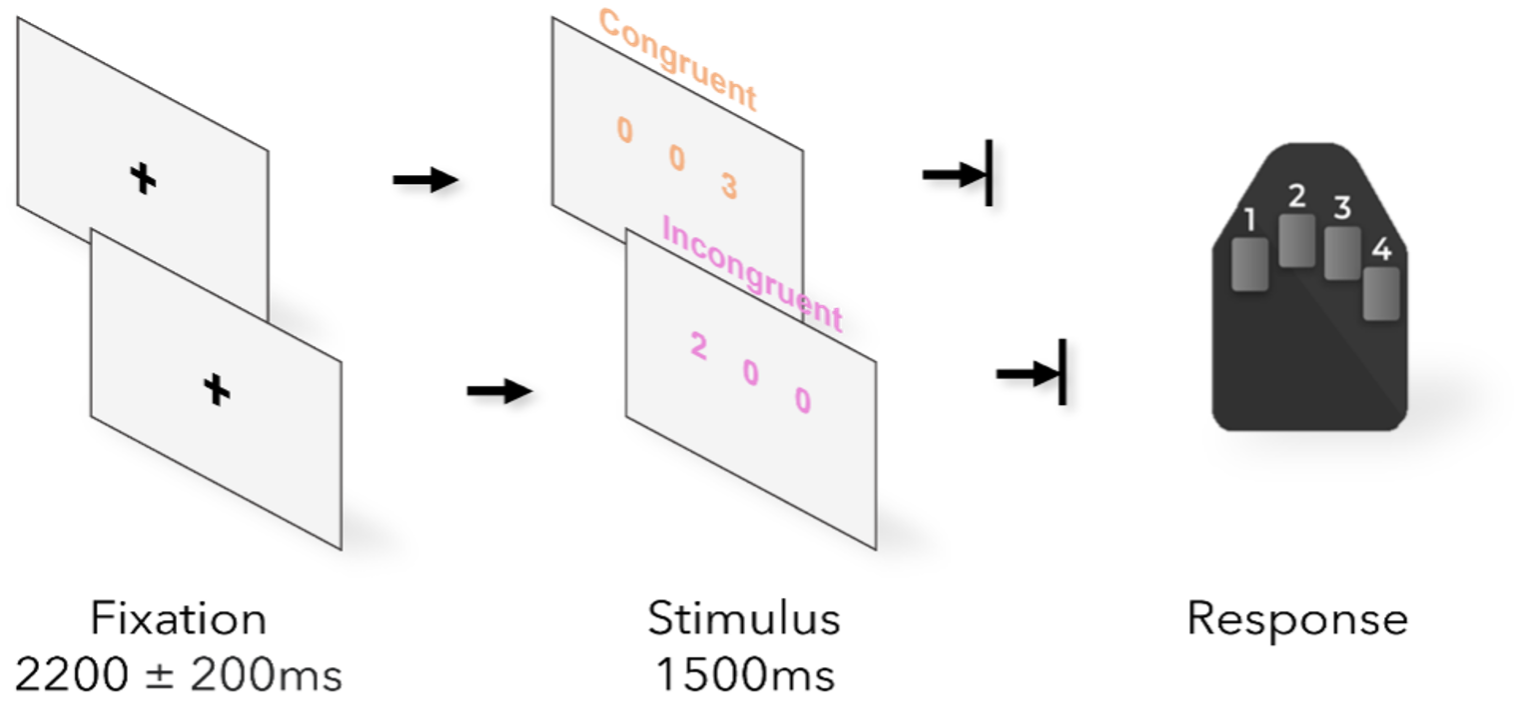
Schematic of the number-based Simon task used in this study. Each participant completed 100 trials of each condition (e.g., control, Simon), which were presented in a counterbalanced block design. Participants indicated the numerical value of the non-zero integer via corresponding button press.

Participants completed a number-based Simon task (Fig. 1) that has been used previously as a stand-alone task and as part of a multi-source interference task (Wiesman & Wilson, 2020a; Wiesman et al., 2020). Custom stimuli were programmed in MATLAB (MathWorks Inc.) using *Psychophysics Toolbox Version 3* (Brainard, 1997) and projected onto a nonmagnetic screen. During MEG recording, participants were asked to focus on a fixation cross displayed in the center of a gray screen, which was presented for 2000–2400 ms. A horizontal array of three equally spaced integers between 0 and 3 then appeared for 1500 ms; two of the integers were identical (task irrelevant), while the third integer was different (i.e., target). On a button pad, where the index, middle, and ring finger corresponded to 1, 2, and 3, respectively, participants had to indicate the numerical identity of the unique (target) integer, *not* its spatial location. Further, youth were told that speed and accuracy were each a crucial part of the task. All participants completed two blocks of 100 trials each: a control condition without interference (e.g., 100/020/003) and the Simon condition, which posed spatial interference (e.g., 010/200/002/030). The two blocks were counter-balanced across participants and the scan time for both conditions combined was approximately 12 minutes, with a 30 second break after 50 trials in each block. Behavioral results were assessed for each participant via computing accuracy as a percentage (correct trials/total trials) and reaction time (RT) for each correct trial. Further, the congruency effect, also termed the Simon effect (Simon & Berbaum, 1990), was calculated by subtracting the mean RT in the control condition from that of the Simon condition per participant.

### 2.6 Structural MRI processing and coregistration

Using the coordinate system established by the cHPI coils and fiducial landmarks, each participant’s MEG data were co-registered with structural anatomy using BESA MRI (Version 2.1; BESA GmbH, Gräfelfing, Germany). Individual structural T1-weighted MR images were used when available (*N* = 107) or alternatively MEG data were fitted to a demographically matched template MRI (*N* = 13) using the scalp surface points prior to source space analyses. Importantly, these two approaches have been shown to yield very similar results (Holliday et al., 2003). Note that the missing MRIs were mainly due to children being afraid of the magnet and/or exhibiting excessive motion during the MRI. Structural MRI data were aligned parallel to the anterior and posterior commissures and transformed into standardized space. Following source analysis (i.e., beamforming), each participant’s 4.0 x 4.0 x 4.0 mm functional images were also transformed into standardized space (Talairach) using the transform that was previously applied to the structural MRI volume and spatially resampled. Peak voxel coordinates were also transformed into MNI space to facilitate comparison with other studies.

### 2.7 MEG preprocessing, time-frequency transformation, and sensor-level statistics

Cardiac and blink artifacts were identified in the raw recordings and removed using signal space projection, which was subsequently accounted for during source reconstruction (Uusitalo & Ilmoniemi, 1997). The continuous magnetic time series was divided into 3500 ms epochs (-2000 to 1500 ms), with the baseline defined as the -500 to 0 ms window prior to stimulus onset. Epochs containing major artifacts (i.e., muscle artifacts, coughs, swallowing) were rejected using an individualized fixed-threshold method, supplemented with visual inspection. Briefly, in MEG, the raw signal amplitude is strongly affected by the distance between the brain and the MEG sensors, as the magnetic field strength falls off sharply as the distance from the current source increases. To account for this source of variance across participants, as well as actual variance in the neural response amplitude, we used individually determined thresholds for all correct trials based on the within-subject signal distribution for both amplitude and gradient to reject artifacts (Ott et al., 2021; Rempe et al., 2023). Following exclusion of 17 participants due to poor behavioral performance (≤ 50% accuracy), the average amplitude threshold for rejecting artifacts across all participants and conditions was 1385.05 (*SD* = 457.71) fT/cm and the average gradient threshold was 240.91 (*SD* = 139.33) fT/(cm*ms). Ultimately, this resulted in an average remaining trial count of 68.34 (*SD* = 3.60) for the control and 67.29 (*SD* = 5.62) for the Simon condition. Given that these trials were high quality and had minimal distortion from artifactual sources, we did not use a fixed group-level threshold for the minimum number of artifact-free trials for inclusion at this stage. Following artifact rejection, we tested whether the number of accepted trials differed between both conditions, and whether the number of accepted trials were associated with age and/or natural log-transformed testosterone values. The paired-sample *t*-test and correlations showed that the number of trials in the control condition did not significantly differ from the Simon condition (*p* > .05) and that the number of accepted trials in each condition were not correlated with age or testosterone (all *p*s > .05).

The artifact-free epochs were next transformed into the time-frequency domain using complex demodulation (Kovach & Gander, 2016) with a time/frequency resolution of 2 Hz/25 ms and a bandwidth of 4–100 Hz. The resulting spectral power estimations per sensor were averaged over trials to generate time-frequency plots of mean spectral density. These sensor-level data were normalized using the respective bin’s baseline power, which was calculated as the mean power during the -500 to 0 ms baseline period. The specific time-frequency windows used for imaging were determined by statistical analyses of the sensor-level spectrograms across all correct trials and the entire array of gradiometers. To reduce the risk of false-positive results while maintaining reasonable sensitivity, a two-stage process was followed to control for type-1 error. In the first stage, paired-sample *t*-tests against baseline were conducted on each data point and the output spectrogram of *t*-values was thresholded at *p* < .05 to define time-frequency bins containing potentially significant oscillatory deviations across all participants. In stage two, the time-frequency bins that survived the threshold were clustered with temporally and/or spectrally neighboring bins that were also below the *p* < .05 threshold, and a cluster value was derived by summing all of the *t*-values of all data points in the cluster. Nonparametric permutation testing was then used to derive a distribution of cluster values, and the significance level of the observed clusters was tested directly using this distribution (Ernst, 2004; Maris & Oostenveld, 2007). For each comparison, 10,000 permutations were computed to build a distribution of cluster values. Based on these analyses, the time-frequency windows that contained significant oscillatory events across all participants and both conditions were subjected to the beamforming analysis.

### 2.8 MEG source imaging and source statistics

Cortical activity was imaged through an extension of the linearly constrained minimum variance vector beamformer known as the dynamic imaging of coherent sources (DICS) beamformer (Gross et al., 2001; Van Veen et al., 1997). This beamformer calculates single images based on the cross-spectral densities of all combinations of MEG gradiometers averaged over the selected time-frequency range and the solution of the forward problem for each location on a grid specified by input voxel space. Following convention, the source power in these images was normalized per participant using a separately averaged pre-stimulus noise period of equal duration and bandwidth (Hillebrand et al., 2005). These images are usually referred to as pseudo-*t* maps, where the units (pseudo-*t*) reflect noise-normalized power differences per voxel between a baseline or passive period and an active task-based period. As stated above, these 4.0 x 4.0 x 4.0 mm functional images were transformed into standardized space using the transform that was applied to the participant’s structural images and spatially resampled. MEG preprocessing and imaging were completed using BESA version 7.0.

Normalized differential source power was computed per condition for the statistically selected time-frequency bands over the entire brain volume per participant. The resulting 3D maps of brain activity were then averaged across participants to assess the neuroanatomical basis of the significant oscillatory responses identified through the sensor-level analyses. To assess neural interference effects, subtraction maps were computed by subtracting the control condition map from the Simon condition map within each participant, per oscillatory response. All beamformer images were visually inspected for significant outliers and were subsequently excluded from whole-brain statistics, separately for each neural response. To investigate the spatially-specific and spectrally-specific effects of testosterone and the interaction between testosterone and sex, we utilized whole-brain analysis of covariance (ANCOVA) models. Because testosterone is known to increase throughout development (Blakemore et al., 2010) and because hormone levels collected through saliva fluctuate throughout the day (Matchock et al., 2007), both variables (e.g., chronological age and time of saliva collection) were included in the model as control variables. This allowed us to examine the unique effects of testosterone above and beyond the effects of age and collection time. Thus, we ran a separate voxel-wise ANCOVA model using SPM12 for each of the obtained subtraction maps of Simon-related interference activity (i.e., per oscillatory response) with age and collection time as continuous variables of no interest, testosterone as a continuous predictor variable, and sex as a categorical predictor, along with the respective testosterone-by-sex interaction term. All ANCOVA maps were thresholded at a cluster-defining significance level of *p* < .005 and corrected for multiple comparisons using a cluster criterion requiring a minimum of at least five contiguous voxels (i.e., ≥320 mm^3^), based on the theory of Gaussian random fields (Poline et al., 1995).

## 3 Results

### 3.1 Demographic data

Of the 120 participants, 17 were excluded due to poor behavioral performance (≤ 50% accuracy) and 16 were excluded due to excessively noisy MEG data (e.g., movement artifacts). The remaining 87 participants had a mean age of 10.44 years (*SD* = 2.39) and included 50 male and 37 female participants. Complete demographic information is presented in Table 1, with the results of independent samples *t-*tests comparing males and females, which indicated no statistical differences by sex.

**Table 1.** Demographic and behavioral results of the final sample.

	Total sample *M* (*SD*)	Males *M* (*SD*)	Females *M* (*SD*)	*p-*value
*N*	87	50	37	-
Age	10.44 (2.39)	10.47 (2.49)	10.39 (2.26)	.77
Testosterone (pg/mL)	34.89 (26.71)	34.84 (28.65)	34.95 (24.24)	.76
Testosterone (ln(pg/mL))	3.28 (0.79)	3.26 (0.79)	3.31 (0.79)	.76
Control RT (ms)	728.75 (151.11)	716.28 (136.76)	730.39 (175.44)	.48
Simon RT (ms)	824.87 (140.40)	814.13 (141.08)	839.30 (139.7)	.43
Simon effect RT (ms)	111.14 (78.17)	111.86 (76.38)	110.43 (80.96)	.94
Control accuracy (%)	94.73% (5.51)	94.71% (6.02)	94.75% (4.82)	.97
Simon accuracy (%)	81.66% (10.13)	81.96% (10.51)	81.28% (9.73)	.75
Simon effect accuracy (%)	13.20% (8.80)	12.93% (8.81)	13.56% (8.89)	.75
Saliva collection time (hr)	15:01 (2:49)	14:54 (2:49)	15:15 (2:58)	.59
Race (W, B/AA, AI/AN, MR)	76, 0, 1, 10	44, 0, 0, 6	32, 0, 1, 4	-
Ethnicity (H, NH)	(3, 84)	2, 48	1, 36	.11

*p*-value reflects independent *t*-test between male and female participants. None were significant.

RT = reaction time, W = white, B/AA = black/African American, AI/AN = American Indian / Alaska Native, MR = mixed race. H = Hispanic, NH = non-Hispanic.

### 3.2 Testosterone and behavioral results

**Fig. 2.**
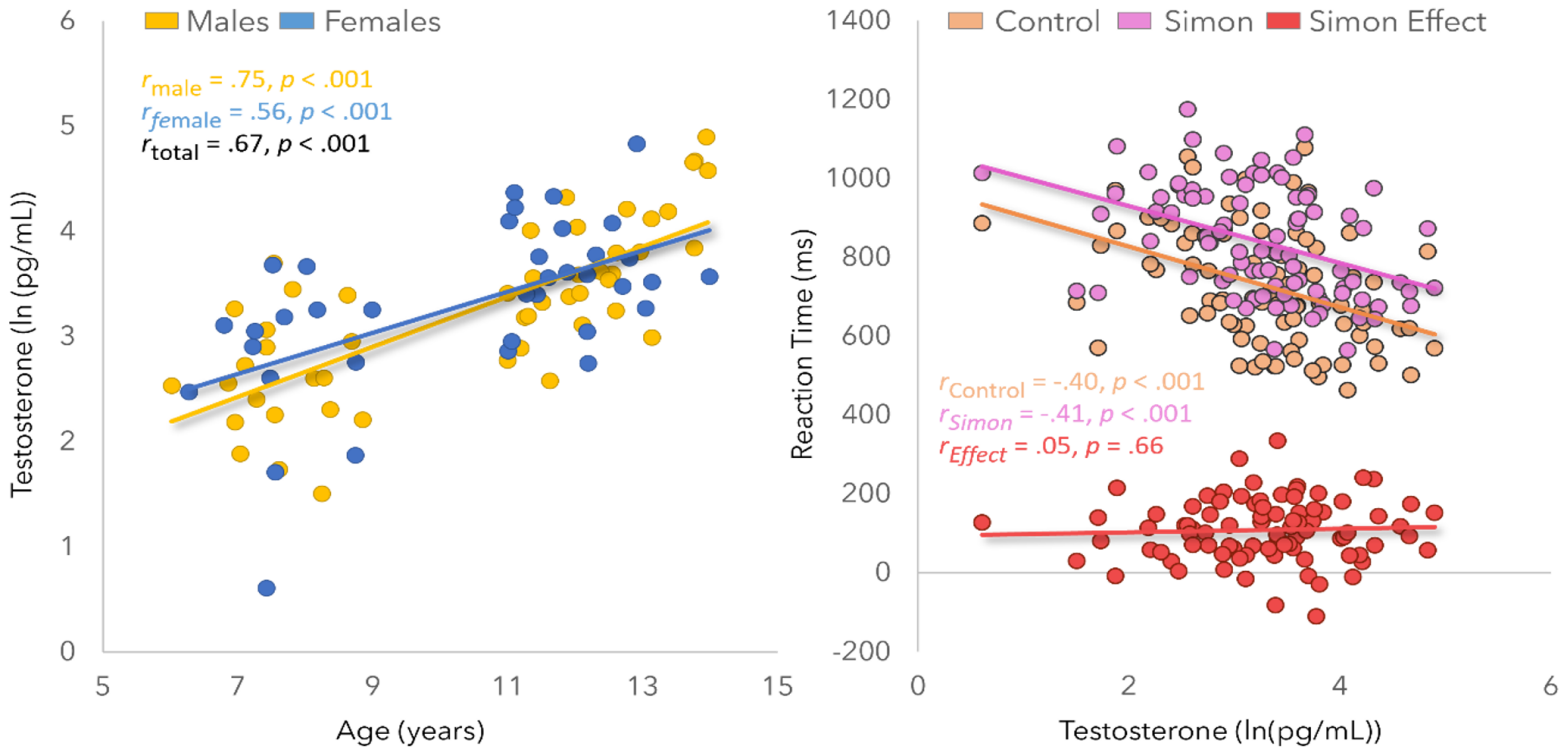
Left: Scatterplot depicting the natural log of testosterone (*y*-axis) as a function of age (*x*-axis), grouped by sex. This relationship was significant in both groups. Right: Scatterplot depicting reaction time (*y*-axis) as a function of the natural log of testosterone (*x*-axis), grouped by condition, with the Simon effect (Simon – Control) shown in red.

Testosterone assays indicated an overall mean of 34.89 pg/mL (males: 34.84 pg/mL; females: 34.95 pg/mL; 1.84 – 133.71) prior to log-transform (Table 1). An independent *t*-test comparing log testosterone levels by sex showed no significant difference [*t*(85) = -.30, *p* = .76]. In contrast, as expected, log testosterone levels were tightly correlated with age (Fig. 2; [*r_t_*_otal_ = .67, *p* < .001; *r*_male_ = .75, *p* < .001; *r*_female_ = .56, *p* < .001]). Task performance was strong, with youths being significantly faster in the control relative to the Simon condition [*t*(85) = -12.99, *p* < .001]. These effects did not differ by sex (Table 1). Lastly, log testosterone levels were correlated with behavioral performance. Specifically, testosterone was negatively correlated with reaction time in the control and Simon conditions, indicating that youths with greater testosterone levels were faster to respond during both conditions [Fig. 2; *r*_control_ = -.40, *p* < .001; *r*_Simon_ = -.41, *p* < .001]. When controlling for age, however, these effects did not remain significant [*r*_control_ = -.13, *p* = .23; *r*_Simon_ = -.09, *p* = .41]. Lastly, there was no significant association between testosterone and the Simon effect [*r*_Simon Effect_ = .05, *p* = .66].

### 3.3 Sensor-level results

**Fig. 3.**
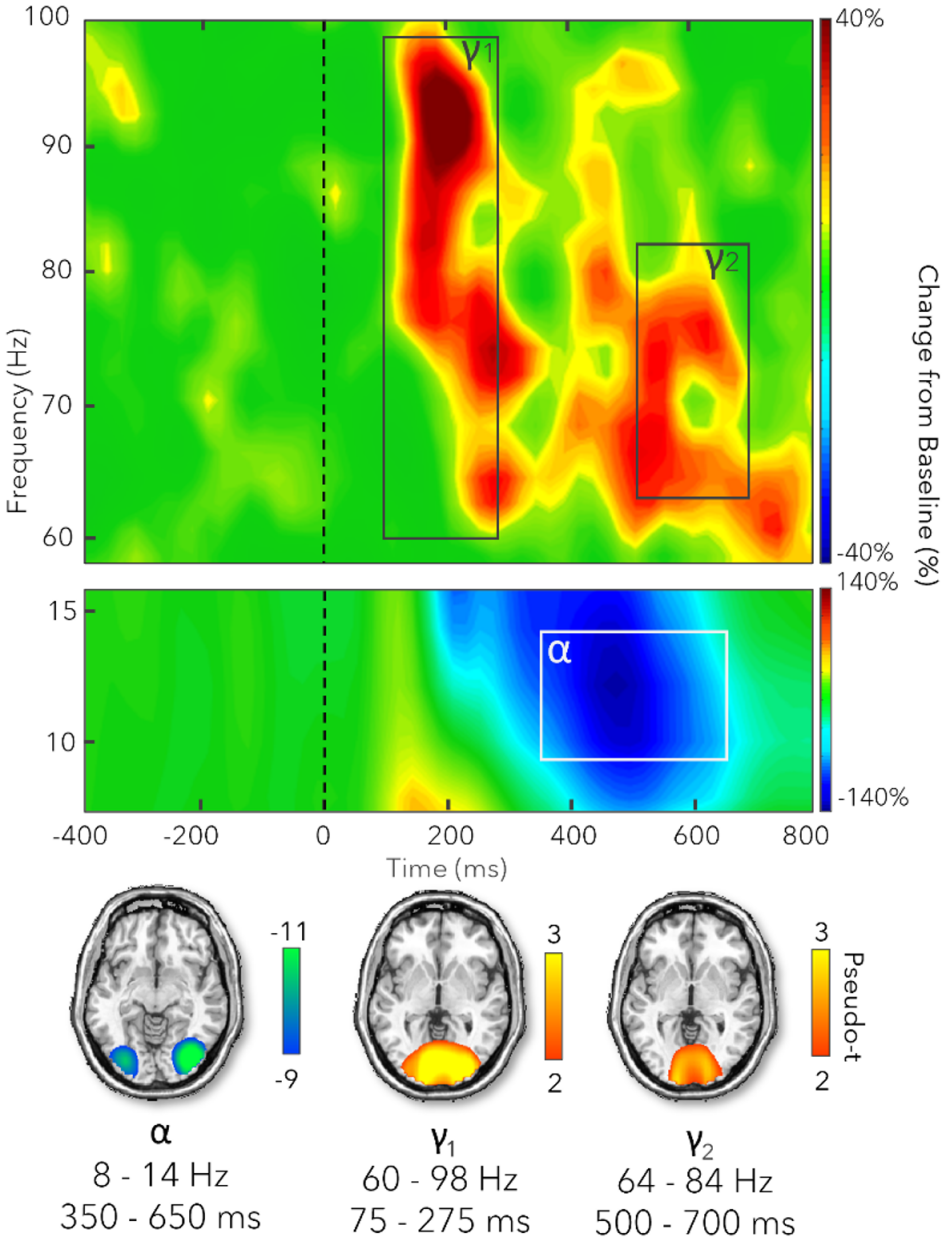
Top: Grand-averaged spectrograms across all participants and conditions for MEG sensors showing the oscillatory responses of interest. Our two-stage approach using permutation testing indicated three time-frequency bins that significantly differed from the baseline period of -500 to 0 ms (*p* < .05, corrected). One alpha window from 350–650 ms (8–14 Hz) was identified, as well as two gamma windows from 75–275 ms (early gamma (γ1): 60–98 Hz) and from 500–700 ms (late gamma (γ2): 64–84 Hz). Bottom: Grand-averaged beamformer images (pseudo-*t*) across all participants and conditions for each time-frequency window.

Sensor-level spectrograms for all correct and artifact-free trials, collapsed across both conditions (i.e., control and Simon), were statistically examined using a two-stage procedure that included nonparametric permutation testing to control for multiple comparisons. The sensor-level data indicated three significant responses (Fig. 3). A significant decrease in power (i.e., desynchronization) within the alpha frequency range (8–14 Hz) was detected from 350–650 ms. In addition, two significant increases in gamma power (i.e., synchronizations) were observed, one ranging from 60–98 Hz during the 75–275 ms window (early gamma) and a second from 64–84 Hz that extended from 500–700 ms (late gamma). Within these time-frequency bins, neural responses were imaged per condition and participant. We next computed Simon interference maps (control maps subtracted from the Simon maps) per time-frequency component to examine the Simon effect on neural activity elicited during the task. These maps were used for the primary analyses of hormone and sex effects.

### 3.4 Main effects of testosterone on oscillatory activity

**Fig. 4.**
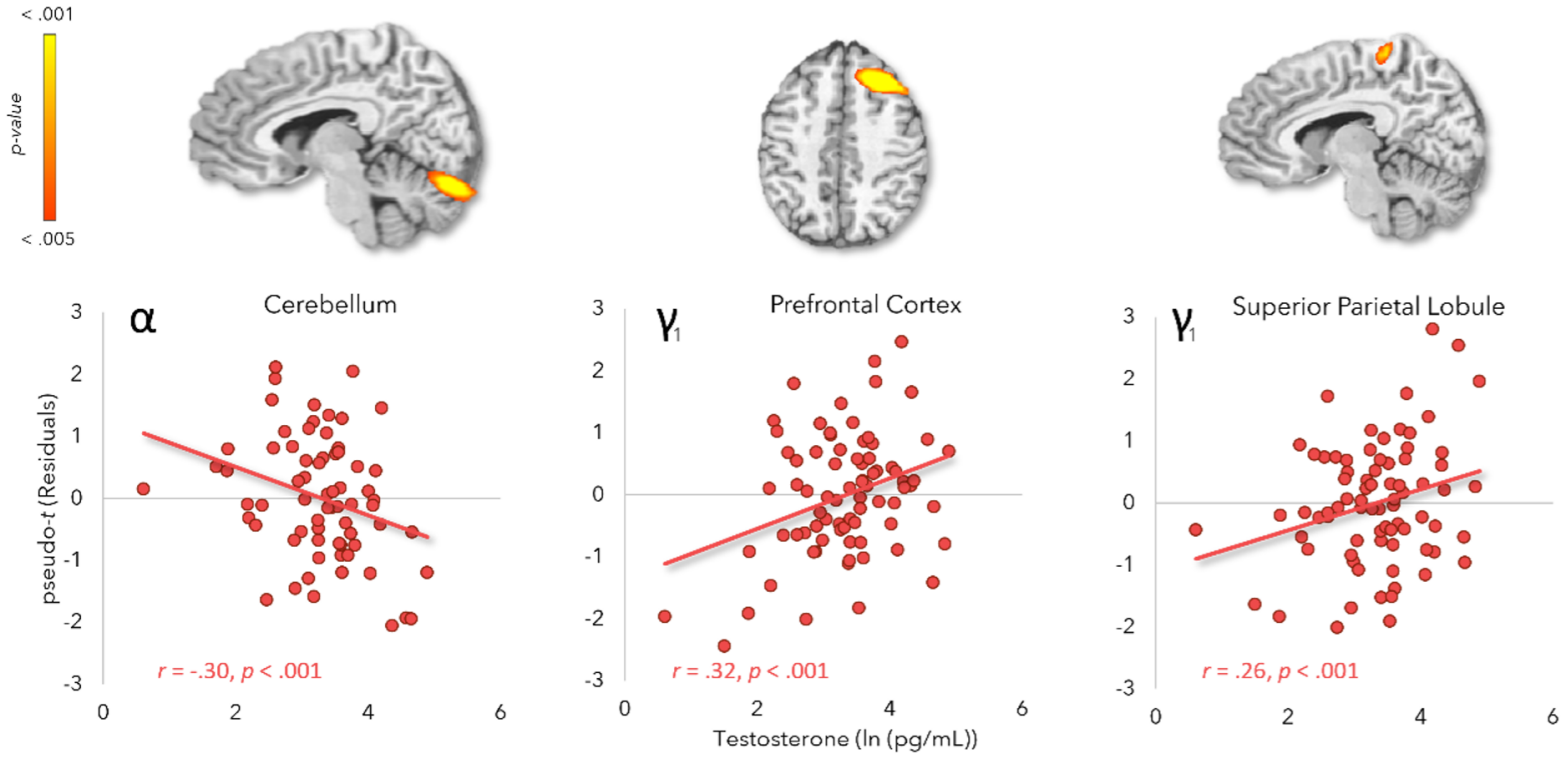
Testosterone main effects on Simon interference maps per oscillatory response based on ANCOVA models with age and saliva collection time as nuisance covariates. Scatterplots depict log-transformed testosterone levels (*x*-axis) as a function of the amplitude of the Simon interference effect (pseudo-*t*) at the peak voxel (*y*-axis). The residual values were calculated by adjusting the pseudo-t values for saliva collection time and age. γ_1_ = early gamma response.

As mentioned previously, the whole-brain participant-specific interference maps (control subtracted from Simon) were subjected to an ANCOVA per significant time-frequency response with log-transformed testosterone and testosterone-by-sex included as effects of interest, while age and saliva collection time were entered as variables of no interest. We subsequently assessed the maps for main effects and interaction effects that survived the threshold of *p* < .005, corrected. In the alpha range, we found one significant main effect of testosterone in the right superior cerebellum [*F*(1,65) = 14.67, *p* = .0003]. This peak exhibited negative directionality, suggesting greater task-related changes in alpha oscillations (i.e., greater decreases in power relative to baseline) during Simon interference as a function of increasing testosterone levels. We also found two main effects of testosterone in the early gamma window; one within the right prefrontal cortex [*F*(1,72) = 16.83, *p* = .0001] and the other in the right superior parietal cortices [*F*(1,72) = 12.11, *p* = .0009]. In both regions, gamma interference responses increased with testosterone levels (Fig. 4). We did not detect any significant effects in the late gamma window.

### 3.5 Testosterone by sex interaction effects on oscillatory activity

**Fig. 5.**
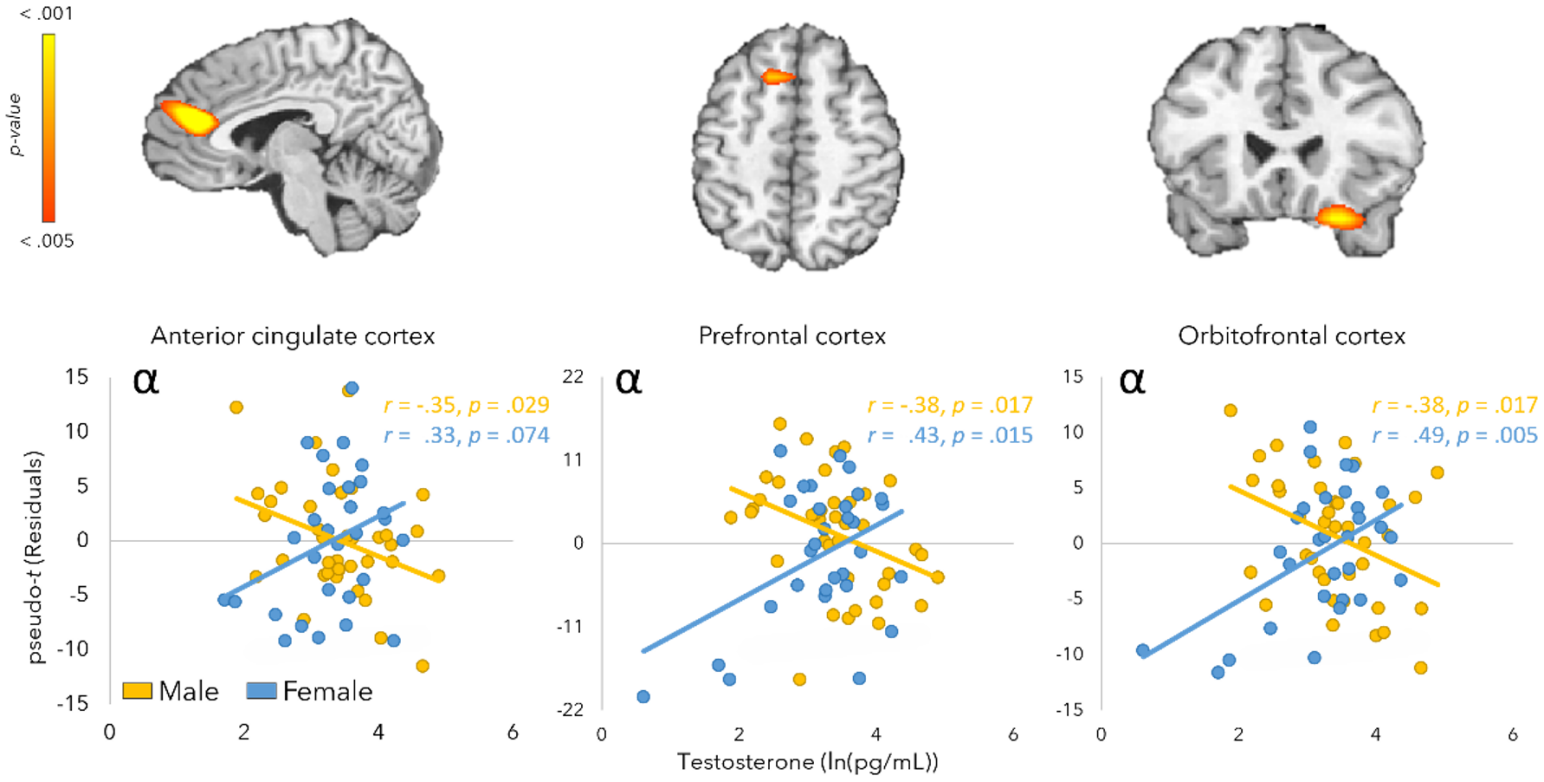
Testosterone-by-sex interaction effects on alpha oscillatory responses. Interaction effects were detected in the whole-brain alpha interference maps in the anterior cingulate, prefrontal, and orbitofrontal cortices. Scatterplots depict log-transformed testosterone levels (*x*-axis) as a function of alpha interference amplitude (pseudo-*t*) at the peak voxel of the interaction effect (*y*-axis). The residual values were calculated by adjusting the pseudo-t values for saliva collection time and age.

Significant testosterone-by-sex interaction effects were detected for the alpha interference responses (Fig. 5). In the anterior cingulate cortex [*F*(1, 64) 16.88, *p* < .001], male participants exhibited a negative relationship between testosterone and alpha interference responses (*r* = -.35, *p* = .029), while female participants exhibited a trending neuro-hormonal effect in the opposite direction (*r* = .33, *p* = .074). However, note that more negative alpha interference responses indicates that alpha oscillations were stronger (i.e., more negative) in the Simon compared to the control condition. Thus, males exhibited stronger Simon interference responses with increasing testosterone. This interaction was also significant in the prefrontal cortex, [*F*(1, 64) 12.28, *p* < .001], where again males had a negative relationship between testosterone levels and alpha interference responses (*r* = -.38, *p* = .017) and females showed the opposite (*r* = .43, *p* = .015). Finally, in the orbitofrontal cortex [*F*(1, 64) 14.09, *p* < .001], the same pattern of a negative relationship between testosterone and alpha interference responses was observed in males (*r* = -.38, *p* = .017), while female participants demonstrated a positive neuro-hormonal effect (*r* = .49, *p* = .005).

**Fig. 6.**
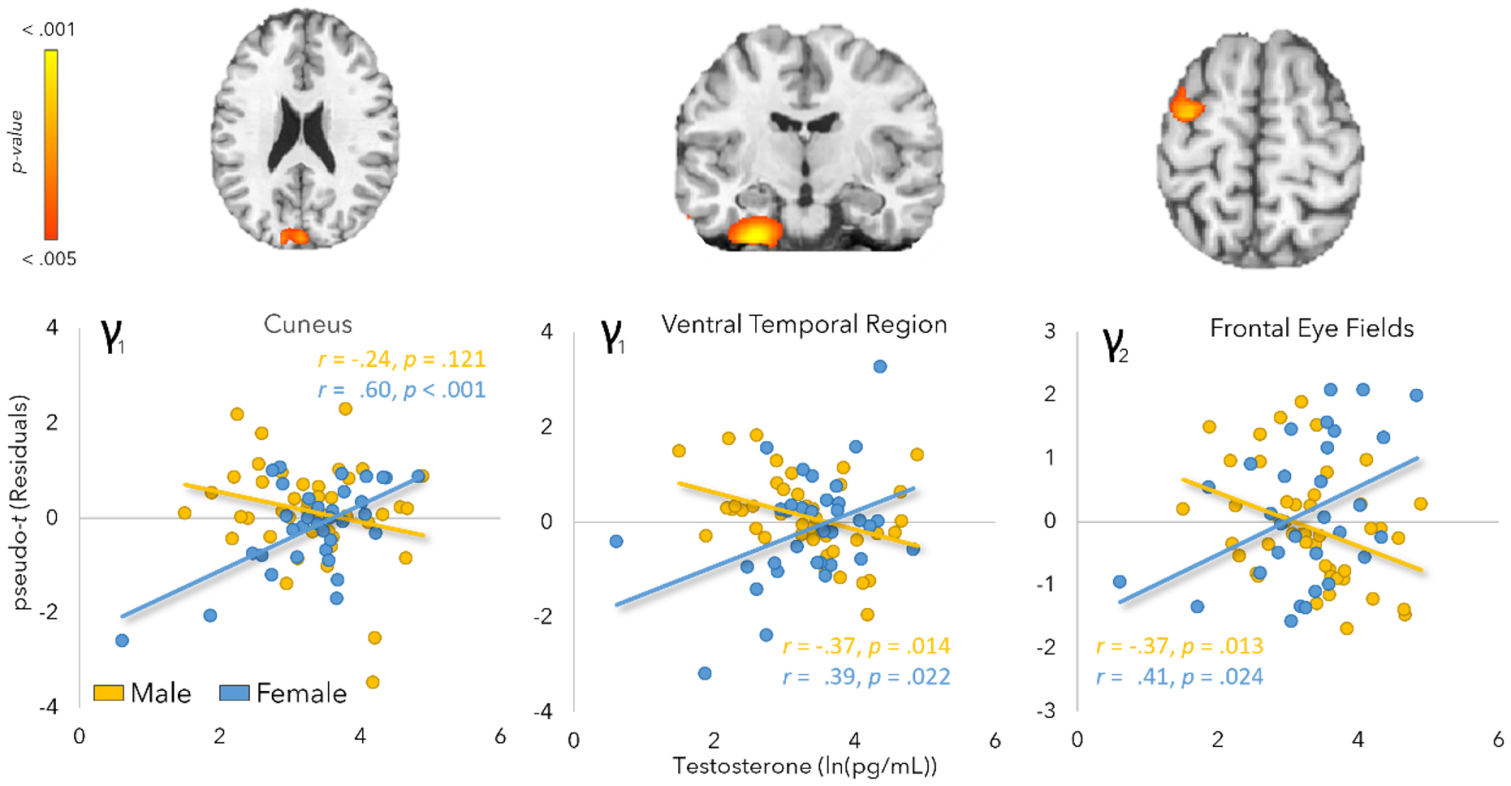
Testosterone-by-sex interaction effects on gamma oscillatory responses. Interaction effects were detected in the whole-brain early and late gamma interference maps. Scatterplots depict log-transformed testosterone levels (*x*-axis) as a function of gamma interference amplitude (pseudo-*t*) at the peak voxel of the interaction effect (*y*-axis). All models were corrected for saliva collection time and age. γ_1_ = early gamma; γ_2_ = late gamma.

Both gamma oscillatory responses (e.g., early and late) revealed significant testosterone-by-sex interaction effects. In the early gamma window, we found a significant peak in the left cuneus [*F*(1,72) = 11.13, *p* = .001], which reflected that males did not exhibit a relationship between testosterone and gamma interference responses (*r* = -.24, *p* = .121) while female participants exhibited a strong positive relationship with gamma interference (*r* = .60, *p* < .001). This indicated that stronger gamma responses in the Simon compared to the control condition was associated with higher testosterone levels in females. In addition, a testosterone by sex interaction was detected in the ventral temporal region [*F*(1,72) = 12.88, *p* = .0006], such that male participants exhibited a negative relationship between testosterone and gamma interference responses (*r* = -.37, *p* = .014) whereas females again exhibited a positive relationship with gamma interference (*r* = .39, *p* = .022). Within the late gamma window, we found a significant interaction peak within the left frontal eye field (FEF; [*F*(1,71) = 11.75, *p* = .001]), where female participants again had a positive relationship between testosterone and gamma interference responses (*r* = .41, *p* = .024) and males exhibited the opposite pattern (*r* = -.37, *p* = .013; Fig. 6). Peak voxel coordinates for all results, including model statistics, are listed in Table 2.

**Table 2.** Coordinates of the peak response in each significant neural Simon interference cluster.

Region of interest	Frequency	X	Y	Z	*F*	*p*
*Testosterone main effect*						
R cerebellum	α	6	-84	-18	14.67	<.001
R PFC	γ^1^	26	26	45	16.83	<.001
R SPL	γ^1^	9	-42	74	12.11	<.001
*Testosterone-by-sex interaction effect*						
R ACC	α	11	42	24	16.88	<.001
L PFC	α	-11	18	51	12.28	<.001
R OFC	α	27	29	-25	14.09	.001
L cuneus	γ^1^	-14	-94	26	11.13	.001
L ventral Temporal	γ^1^	-10	-4	-34	12.88	<.001
L FEF	γ^2^	-34	-13	69	11.75	.001

*Note*: All coordinates are in MNI space.

PFC = prefrontal cortex; SPL = superior parietal lobule; FEF = frontal eye field; OFC = orbitofrontal cortex; ACC = anterior cingulate cortex.

## 4 Discussion

The present study examined the impact of developmental increases in testosterone levels on the neural oscillatory dynamics serving selective attention in typically developing youth. Overall, participants performed well on the task, and those with higher testosterone levels generally had faster responses than those with lower testosterone levels, although this effect did not persist once controlling for age. We reproduced the classic Simon interference effect, whereby participants responded faster during the control condition compared to the Simon condition. We also observed robust oscillatory activity in the alpha (8–14 Hz) and gamma (60–98 Hz; 64–84 Hz) bands during task performance and identified novel spectral- and sex-specific responses that were associated with changes in testosterone. Specifically, our key findings were that neural interference effects in the alpha and gamma range became stronger with increasing testosterone while controlling for age and saliva collection time. Additionally, we found that the associations between rising testosterone and alpha and gamma oscillations differed by sex in brain regions crucial for attention function, including the prefrontal, cingulate, and parietal cortices. Below, we discuss the implications of these findings.

### 4.1 Spectrally-specific relationships among testosterone and neural attention function

Our main findings were that testosterone levels were significantly associated with Simon interference-related responses in the alpha and early gamma bands, and these results were observed above and beyond the effects of age, sex, and saliva collection time. Within the alpha band, a stronger interference response (i.e., a stronger decrease in power from baseline) was found in the right cerebellum as a function of increasing testosterone. The cerebellum is thought to modulate predictive coding during attention by reallocating attentional resources in early visual areas and is thought to contribute crucially to timing and processing order in the cortex (Kellermann et al., 2012; Schlerf et al., 2012). Other work has shown that alpha oscillations are crucial to the inhibition of irrelevant stimuli (Clayton et al., 2018; Forschack et al., 2017; Fries et al., 2001; Gutteling et al., 2022; Heinrichs-Graham & Wilson, 2015; Jensen & Mazaheri, 2010; Wilson et al., 2017; Womelsdorf & Fries, 2007) and have been identified previously in cerebellar regions upon visual stimulus presentation (Heinrichs-Graham & Wilson, 2015; McDermott et al., 2017; Taylor et al., 2021). Further, modulation of sex steroid binding sites in the cerebellum has been linked to alterations in gray matter volume and supratentorial functions (Schutter et al., 2017). Taken together, testosterone may be an important marker of increased cerebellar recruitment and reallocation of inhibitory signaling throughout development.

We also observed a significant main effect of testosterone in the early gamma window with increased neural interference in the right prefrontal cortex and right superior parietal cortices. Both regions have previously been identified in selective attention processing (Lew et al., 2018; McDermott et al., 2017; Taylor et al., 2021) as part of the frontoparietal attention network (Rosenberg et al., 2021). On a cellular level, gamma oscillations are highly influenced by GABAergic interneurons synapsing on excitatory pyramidal cells, which, in turn, leads to increased efficiency in neural processing during attention (Bartos et al., 2007; Buzsáki, 2010; Buzsáki & Wang, 2012; Edden et al., 2009; Fries et al., 2007). GABA receptors have been shown to be impacted directly by testosterone, increasing receptor density (Hebbard et al., 2003) and affinity to agonists (Aikey et al., 2002; Laube et al., 2020; McHenry et al., 2014; Reddy & Jian, 2010), suggesting that testosterone may have a direct impact on neural oscillatory dynamics by way of modulating GABA receptors. Our results indicate that increases in testosterone through a pubertal transition period are associated with changes in gamma oscillatory activity, though more research is needed to interrogate the interplay between GABA and testosterone.

### 4.2 Sex-by-testosterone oscillatory responses

Sex-by-testosterone effects were found in the alpha and gamma time windows. Specifically, sex-by-testosterone interaction effects were detected in the anterior cingulate, prefrontal, and orbitofrontal cortices within the alpha spectral range. The anterior cingulate cortex (ACC) and prefrontal cortex (PFC) have been consistently identified in various tasks probing selective attention and are known to follow a protracted developmental course (Bunge et al., 2002; Friedman & Robbins, 2022; Hwang et al., 2016; Luna et al., 2010; McDermott et al., 2017; Sheridan et al., 2014; Son et al., 2024; Taylor et al., 2021). The ACC plays a critical role in conflict and performance monitoring, while the PFC broadly exerts top-down modulation of posterior sensory cortices (Badre & Wagner, 2004; Kerns, 2006; McLaughlin et al., 2019; Sheridan et al., 2014). Interestingly, prior studies have identified hormone-related sex differences in cortical thinning in the ACC (Koolschijn et al., 2014); taken together, these results suggest that both the structure and function of higher-order structures involved in selective attention are sensitive to the effects of pubertal hormones in a sex-dependent manner. Furthermore, sex differences in neural recruitment have previously been identified in higher-order regions, including the PFC, despite comparable behavioral performance across many (but not all) tasks (Gaillard et al., 2021; Goldstein et al., 2005). Similarly, we did not observe sex differences in behavioral performance in this sample, though sex differences in neural function were identified throughout the cortex. While sex differences in brain structure have been consistently noted, the literature focusing on sex differences in brain function and their subsequent contributions to behavioral performance differences has been much more heterogeneous. Of note, neural differences are not always accompanied by differences in behavior, which has been interpreted in the literature as divergent, but equally effective means of task performance. Taken together, our results suggest that these sex-dependent relationships between testosterone levels and oscillatory activity may reflect alternative strategies of processing that are equally effective and begin to emerge earlier in girls due in part to hormonal processes (Grissom & Reyes, 2019; Shansky, 2018; Shansky & Murphy, 2021).

In addition, we identified sex-by testosterone effects within the early gamma window in the cuneus and ventral temporal region, with females exhibiting increased gamma interference responses with rising testosterone and male participants showing the opposite pattern. Prior work has linked the cuneus to early visual processing (Gao et al., 2013; Hahn et al., 2006; Vanni et al., 2001), while the ventral temporal cortex is known to play a crucial role in the categorization of visual responses and is an essential node in the ventral visual stream (Grill-Spector & Weiner, 2014). We also found sex-by-testosterone effects within the late gamma window, with female participants again exhibiting stronger gamma interference responses with increasing testosterone in the left frontal eye fields (FEF) and male participants having the opposite pattern in this brain region. The FEF is a component of the dorsal attention network (Vossel et al., 2014) and likely interacts with the visual streams (e.g., striate cortex, ventral visual system) to facilitate feature detection and attention allocation in this task. Overall, testosterone may be a facilitating factor in the development of sexually divergent brain networks (Durdiakova et al., 2011; Herlitz et al., 2013). Our findings build upon and extend a growing body of literature finding sexually-divergent trajectories of neurophysiological patterns serving several cognitive domains associated with pubertal hormones (Fung, Heinrichs-Graham, et al., 2022; Fung, Rahman, et al., 2022; Fung et al., 2020; Killanin et al., 2023; Penhale et al., 2022; Picci et al., 2023), and add novel task-based associations between testosterone and alpha and gamma oscillatory dynamics.

### 4.3 Limitations

Before closing, it is important to discuss the limitations associated with the current investigation. First, our analyses utilized saliva-based measurements of hormone levels, which are sensitive to diurnal changes in hormone concentrations. While we mitigated these effects by controlling for the time of day at which saliva samples were collected, future studies could aim to improve precision by collecting hair samples and/or standardizing sample collection time. Second, future work should examine whether other pubertal hormones (e.g., estradiol, DHEA) have convergent or differing effects during selective attention tasks, as regions elicited in this task are rich with androgen receptors (e.g., cerebellum). Moreover, integrating measures of pubertal status, such as the Pubertal Development Scale, would garner a more comprehensive picture of how pubertal processes affect continued refinement of higher-order cognition during development. Third, this cross-sectional analysis would be strengthened by utilizing a longitudinal design, allowing for a more nuanced investigation that parses within-subject effects and between-subject factors in a developmental context. Finally, our investigation did not have equal representation across the full spectrum of development and future studies should enroll both younger and older typically developing children and adolescents.

### 4.4 Conclusion

The current study identified novel developmental links between testosterone and selective attention function in the alpha and gamma ranges. Our study provides novel insight on the impact of testosterone on the development of neural oscillatory dynamics serving selective attention. We found that testosterone levels were associated with increases in both alpha and gamma Simon interference responses during task performance in key brain regions for attention function. Additionally, we identified sexually divergent effects in regions despite comparable behavioral performance, which may indicate that such neural differences begin to emerge early (i.e., prior to the appearance of secondary sex characteristics) and are at least partially attributable to testosterone differences. These sexually dimorphic oscillatory responses may reflect the activational effects of sex hormones that modulate pre-existing organizational differences, leading to divergent but equally effective selective attention faculties. Further, it is possible that these differences are driven in part by differences in pubertal onset, beginning 1 to 2 years earlier in female children compared to their male counterparts. Thus, while studies of selective attention in youth have primarily focused on chronological age, our results indicate the importance of incorporating hormonal information in developmental studies.

## Data Availability

The data used in this article will be made publicly available through the COINS framework upon the completion of the study (https://coins.trendscenter.org/).
